# Epigenetic regulation of Runx2 transcription and osteoblast differentiation by nicotinamide phosphoribosyltransferase

**DOI:** 10.1186/s13578-017-0154-6

**Published:** 2017-05-23

**Authors:** Min Ling, Peixin Huang, Shamima Islam, Daniel P. Heruth, Xuanan Li, Li Qin Zhang, Ding-You Li, Zhaohui Hu, Shui Qing Ye

**Affiliations:** 1 Department of Pediatrics, Children’s Mercy, 2401 Gillham Road, PRC/4th FL, Kansas City, MO 64108 USA; 2 Department of Biomedical and Health Informatics, University of Missouri Kansas City School of Medicine, Kansas City, MO USA; 3 Spinal Surgery Division, The People’s Hospital of Liuzhou, Guilin Medical University, 8 Wenchang Road, Liuzhou, 545006 Guangxi Province China

**Keywords:** Nampt, Runx2, Osteoblasts, Bone marrow stromal cells, Acetyl-Histone H3 (Lys9)

## Abstract

**Background:**

Bone degenerative disorders like osteoporosis may be initiated by age-related shifts in anabolic and catabolic responses that control bone homeostasis. Although there are studies suggesting that metabolic changes occur with stem cell differentiation, the molecular mechanisms governing energy metabolism and epigenetic modification are not understood fully. Here we reported the key role of nicotinamide phosphoribosyltransferase (Nampt), which is the rate-limiting enzyme in the salvage pathway of NAD biosynthesis from nicotinamide, in the osteogenic differentiation of bone marrow stromal cells.

**Results:**

Differentiated bone marrow stromal cells isolated from *Nampt* ^+/−^ mice presented with diminished osteogenesis, as evaluated by alkaline phosphatase (ALP) staining, ALP activity and osteoblast-mediated mineralization, compared to cells from *Nampt* ^+/+^ mice. Similar results were observed in differentiated Nampt-deficient C3H/10T1/2 and MC3T3-E1 cells. Further studies showed that Nampt promotes osteoblast differentiation through increased function and expression of *Runx2* as tested by luciferase reporter assay, RT-PCR, and Western Blotting. Our data also demonstrated that Nampt regulates *Runx2* transcription in part through epigenetic modification of H3-Lys9 acetylation.

**Conclusion:**

Our study demonstrated that Nampt plays a critical role in osteoblast differentiation through epigenetic augmentation of *Runx2* transcription. NAMPT may be a potential therapeutic target of aging-related osteoporosis.

## Background

Bone loss is a common characteristic of aging and with a world-wide increase in an older population, osteoporosis has become a global health problem in terms of both increased medical costs and decreased quality of life. To maintain bone density and integrity, complex networks and numerous interactions occur between different bone cell types and their environment [1, 2]. Bone is constructed through 3 processes: osteogenesis, modeling, and remodeling. All these processes are mediated by osteoblasts, which synthesize the bone extracellular matrix (osteogenesis) and work in tight coordination with bone-resorbing osteoclasts [3]. Recent emerging evidence demonstrated that osteoblasts and adipocytes originate from common mesenchymal precursor cells. Osteoblast development is governed by the activation of Wnt/β-catenin signaling and the expression of several master transcription factors, including the Runt-related transcription factor (Runx2) [4–7].

Runx2 is required for the expression of multiple osteogenic genes, including collagen I, osteopontin, alkaline phosphatase (ALP), bone sialoprotein and osteocalcin [8]. Runx2 functions by binding to regulatory sites in osteogenic gene promoters in order to activate transcription. In vitro studies show that Runx2 expression is regulated at multiple levels during osteoblast differentiation, including transcription, mRNA stabilization and translation [9–11].

Nicotinamide phosphoribosyltransferase (NAMPT), also known as pre-B cell colony-enhancing factor (PBEF) and visfatin, is the rate-limiting enzyme for NAD^+^ biosynthesis of a mammalian salvage pathway from nicotinamide [12]. The intracellular levels of NAD^+^ and nicotinamide have recently been linked to osteogenesis, suggesting a possible mechanism for the development of senile osteoporosis [13]. These response pathways include promoting the activity of SIRT1, a member of the sirtuin family of NAD-dependent deacetylases [14]. Our previous study also demonstrated that resveratrol, which is a SIRT1 activator, could exert anti-aging effects by increasing telomerase reverse transcriptase (TERT) through elevating NAMPT and intracellular NAD+ levels [15]. Overexpression of NAMPT has been shown to increase SIRT1 activity [12]. Age-related reduction of NAMPT has also been linked to increased adipogenesis [13]. Although these observations provided the correlation of Nampt to the lineage fate determination of mesenchymal stem cells (MSCs), the molecular mechanism by which Nampt regulates osteogenic differentiation in bone marrow stromal cells has not been elucidated.

In this study, we tested osteoblast formation in differentiated bone marrow stromal cells isolated from both *Nampt* wild-type (*Nampt* ^**+/+**^) and *Nampt* heterozygous (*Nampt* ^**+/−**^) mice. Our results indicated that in differentiated bone marrow stromal cells isolated from heterozygous mice, the osteogenic differentiation was lower than those derived from wild-type mice. Further investigation in osteoblasts identified that in Nampt-deficient cells, or in Nampt activity-inhibited cells, osteoblast differentiation was inhibited. Additional investigations also suggested that age-related Nampt reduction could inhibit *Runx2* transcriptional activity and expression, and consequently decreased osteogenesis in bone marrow stromal cells.

## Methods

### Cell and mouse bone marrow stromal cell culture

The murine fibroblast C3H/10T1/2 Clone 8 (CCL-226™) and preosteoblastic MC3T3-E1 Subclone 24 (CRL-2595™) were obtained from the American Type Culture Collection (ATCC^®^, Manassas, VA, USA). The cells were cultured in Modified Eagle’s Medium alpha (α-MEM, Catalog#: A10490, Life Tech., Grand Island, NY, USA) supplemented with 10% fetal bovine serum (Catalog#: S11150, Atlanta Biologicals, Flowery Branch, GA, USA), and 1% of penicillin/streptomycin (Catalog#: 15140-122, Life tech.) at 37 °C in a humidified 5% CO_2_ atmosphere. For osteoblast differentiation, cells were cultured in osteoblast medium (OBM), including α-MEM medium supplemented with 10% FBS, 10 mM β-glycerophosphate (Catalog#: 251291, Sigma, St Louis, MO, USA), 50 µg/mL ascorbic acid (Catalog#: A5960, Sigma) and 0.1 µM dexamethasone (Catalog#: D4902, Sigma) for the indicated days with medium changes twice a week.

Mouse bone marrow stromal cells were obtained from 6- to 8-week-old male C57BL/6 wild-type *Nampt* ^+/+^ and *Nampt* ^+/−^ mice generated as described previously [16]. Briefly, mice were euthanized using 4% isofluorane in CO_2_, and the bones were excised aseptically from the hind limbs. External soft tissue was discarded, and the bones were place in a-MEM supplemented with 1% penicillin/streptomycin. Both ends of the femur and tibia were clipped. An 18-gauge needle was inserted into the diaphysis at one end, and bone marrow was flushed out from the other end to a 50-mL Falcon tube by culture medium. After centrifugation at 1000 rpm for 5 min, the cell pellet was collected and diluted in 15 mL of culture medium and cultured in a 75-cm flask. Non adherent cells were removed after 24 h, and the remaining cells were passaged after reaching 80% confluence. For osteoblast differentiation, cells were cultured in OBM for 2 weeks, with medium changes twice per week. All mouse experiments were conducted in accordance with NIH guidelines and were approved by the University of Missouri Kansas City Animal Care and Use Committee.

### Gene transfection of murine mesenchymal stem cell line MC3T3-E1

Briefly 2 × 10^5^ cells/well were seeded into 6-well plates and incubated overnight, then exposed to mouse Nampt shRNA (Catalog#: CSTVRS TRCN0000101275, Sigma) or pLKO.1 non-mammalian shRNA control lentiviral particles (Catalog#: SHC002H, Sigma) with 8 µg/mL of polybrene for 24 h. Following the transduction, cells were selected with 800 ng/mL puromycin (Catalog#: ant-pr-1, InvivoGen, San Diego, CA, USA) for 7 days. Puromycin resistant, stably transfected cells were used for further experiments.

### Alkaline phosphatase (ALP) enzyme staining and quantification, alizarin red staining

Staining of ALP activity was performed with BCIP/NBT substrate solution (Catalog#: B1911, Sigma Aldrich), according to the manufacturer’s instructions.

Calcium deposition was visualized by alizarin red S (catalog#: A5533, Sigma Aldrich) staining [13]. Cells were cultured in 24-well plates for 2 weeks in OBM, fixed in ice-cold 70% ethanol for 60 min, and incubated with alizarin red (2%, pH 4.2) for 10 min at room temp prior to microscopy. An average of 200 cells/well were counted to calculate the percentage of ALP and alizarin red positive cells.

PNPP (p-nitrophenyl phosphate disodium salt, Catalog#: 34045, Pierce, Rockford, IL, USA) was used to quantify the ALP activity in cell cultures [13]. Cells were plated at 20,000/well in 6-well plates and cultured in OBM for 4 days. The cells were lysed in 500 µL of M-PER mammalian protein extraction reagent without protease inhibitors (Catalog#: P8340, Sigma), followed by incubation (20 µL lysate) with 100 µL of PNPP solution in 96-well plate at room temperature for 30 min. Then 50 µL of 2 N NaOH was added to stop the reaction. The blank control was 20 µL of M-PER reagent and 100 µL of PNPP solution. The absorbance was measured at 405 nm in a kinetics ELISA reader (BioTek, Winooski, VT, USA). The results were normalized with the protein concentration of the cell lysates.

### Isolation of RNA, qPCR, Western blot, and NAD/NADH analysis

Total RNA was isolated from MC3T3-E1 cells with a mirVana™ miRNA Isolation Kit (Catalog#: AM1561, ThermoFisher Scientific, Waltham, MA, USA) according to the supplier’s instructions. RT-PCR was performed with the cDNA synthesis catalyzed by Superscript III (Catalog#11752-250, ThermoFisher) and PCR amplification using *Runx2* specific primers (Forward: 5′-CCCAGCCACCTTTACCTACA-3′, Reverse: 5′-TATGGAGTGCTGCTGGTCTG-3′) synthesized by Integrated DNA Technologies (IDT, Coralville, IA, USA).

Western blots were performed as described previously [17]. Briefly, an equal amount (20 µg) of protein per each sample was analyzed by SDS polyacrylamide gel electrophoresis and transferred to PVDF membrane. The membrane was incubated with Anti-Nampt antibody (Catalog#: AG-20A-0034, Santa Cruz Bio., Santa Cruz, CA, USA; 1:3000) overnight at 4 °C with gentle shaking. The immune complex was detected with a 1:4000 dilution HRP conjugated anti rabbit secondary antibody. Gapdh (Catalog#: sc-25778, Santa Cruz Bio.) was detected as the loading control.

NAD/NADH assays were performed using an Amplite™ fluorimetric total NAD/NADH assay kit (Catalog#: 15257, AAT Bioquest, Sunnyvale, CA, USA) according to the manufacturer’s instruction. 10 µg protein for each sample was applied for the assay.

### Chromatin immunoprecipitation (CHIP) assay and luciferase reporter assay

Chromatin immunoprecipitation (ChIP) assays were performed using a Simple ChIP Enzymetic Chromatin IP Kit (Catalog#: 9003, Signaling tech. Beverly, MA, USA) following the manufacturer’s protocol. 4 × 10^6^ cells were used for each reaction. Histone acetylation was determined by using specific antibodies against acetylated histone H3 at lysine 9 (K9). Immunoprecipitated DNA was reverse cross linked, purified and analyzed by PCR for 32 cycles. PCR primers were designed upstream of the *Runx2* transcriptional start site (TSS) (mRunx2-155-Forward: 5′-AGAAAGAGGGAGGGAAGAGAGC-3′, mRunx2 +30-Reverse: 5′-TTGTTTGTGAGGCGAATGAAGC-3′).

Functional analyses of the *Runx2* promoter were performed using the Dual-Glo Luciferase Assay System (Catalog#: E1910, Promega, Madison, WI, USA). The *RunX2* promoter region (−3471 to +390) [18] was PCR amplified (mRunX2 −3471F: 5′-CCGGTACCTTTGCTAACACAGAACAATTTCACG-3′; mRunX2 +390R: 5′-CCCTCGAGCAGATAGAACTTGTGCCCTCTGTT-3′) from mouse genomic DNA and cloned into the *Kpn*I and *Xho*l sites of the pGL4.10-Basic luciferase reporter vector (Catalog#: AY738222, Promega, Madison, WI, USA). The recombinant pGL4.10-*Runx2*pro constructs were verified by sequencing. To determine Nampt’s role in the transcriptional regulation of *Runx2*, differentiated MC3T3 cells (48 h) were co-transfected with pGL4.10-*RunX2*pro (100 ng/well), pGL4.75 Renilla luciferase control (Plasmid #44571, Addgene) plasmid (4 ng/well), and either 100 µM Nampt siRNA (ThermoFisher Scientific) or 100 µM scrambled siRNA control with Lipofectamine 3000 (Catalog#: L3000015, ThermoFisher) and cultured in 96 well plates at a density of 2.5 × 10^4^ cells/well for an additional 24 h. Luminescence was measured and analyzed in accordance with the manufacturer’s instructions on a TriStar LB 941 Multimode Microplate Reader (Berthold, Bad Wildbad, Germany). Firefly luciferase activities were normalized against Renilla luciferase activities following the subtraction of background luminescence. Relative levels of luciferase activity were normalized against MC3T3 cells transfected with the pGL4.10 empty vector.

### Statistics

Statistical analyses were carried out using the Sigma Stat (ver.4.0, Systat Software, Inc., San Jose, CA, USA). All data were expressed as mean ± SD (standard deviation). Differences among treatments were assessed by the one-way analysis of variance (ANOVA) followed by the Holm-Side post hoc test. Differences between groups were considered statistically significant at p < 0.05.

## Results

### The decreased osteoblast differentiation in bone marrow stromal cells from Nampt deficient mice

**Fig. 1:**
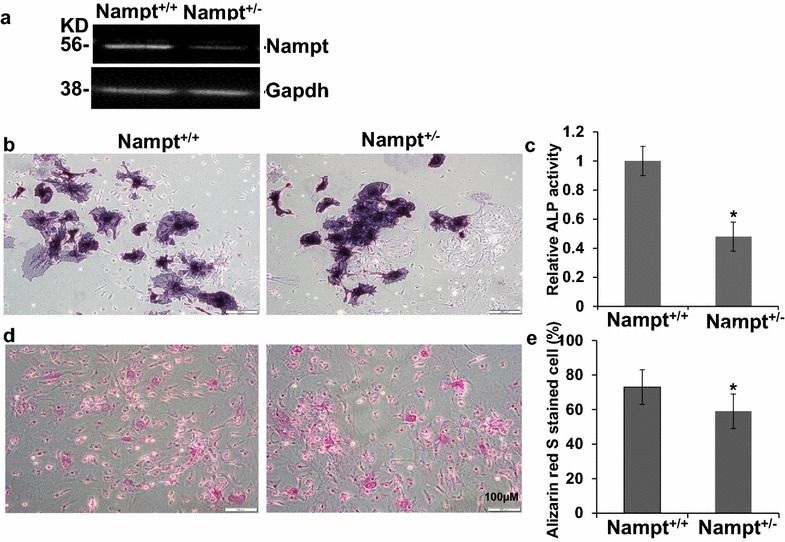
Osteoblast differentiation of bone marrow stromal cells in wild type (*Nampt* ^+/+^) and *Nampt* ^+/−^ mice. Cells were isolated and cultured in 12-well plate in RPMI for 10 days and then were differentiated in OBM for 14 days. **a** Western blot analysis of Nampt expression of differentiated osteoblast in *Nampt* ^+/+^ and *Nampt* ^+/−^ mice. **b** Representative images of ALP stained differentiated osteoblasts. **c** ALP activity analyzed by PNPP quantification described in “Methods”. **d** Representative images of the differentiated osteoblast stained by Alizarin Red S. **e** Alizarin Red S stained differentiated osteoblast were counted using the Image J in three different view fields and the percentage of positive cells over total cells were calculated. n = 3, *p < 0.05 vs wild type mice

To investigate the role of Nampt in osteogenesis in bone stromal cell differentiation, bone morrow cells were isolated from C57BL/6J *Nampt* wild type (*Nampt* ^+/+^, n = 3), or *Nampt* heterogeneous mice (*Nampt* ^+/−^, n = 3), and were cultured in OBM medium for 14 days. Western blotting demonstrated that Nampt expression in the differentiated stromal cells derived from *Nampt* ^+/−^ mice was lower than those derived from wild-type mice (Fig. 1a), suggesting the concordance between genotype and phenotype in *Nampt* ^+/−^ mice. The ALP stained cells, a biomarker of osteoblasts, were also significantly less in cells derived from *Nampt* ^+/−^ mice than that derived from wild-type mice (Fig. 1b). To confirm the ALP staining results, ALP activity quantification was performed. ALP activity in cells isolated from *Nampt* ^+/−^ mice was significantly lower (0.48 ± 0.02) than those from wild-type mice (Fig. 1c).

Osteoblast-mediated mineralization is indicative of the formation of bone mass and can be detected specifically using Alizarin Red S. The bone marrow stromal cells derived from heterozygous *Nampt* ^+/−^ mice had significantly lower Alizarin Red S stained cells than those from wild type *Nampt* ^+/+^ mice (Fig. 1d). Alizarin Red S staining also showed that 73% were positive in bone morrow stromal cells derived from *Nampt* wild type mice, while only 59% of cells from *Nampt* ^+/−^ mice were positive, which was significantly lower than those from wild-type mice (p = 0.037).

The above data collectively suggested that in wild-type mice, bone morrow stromal cells were more readily differentiated into osteoblasts than those derived from Nampt deficient mice, suggesting a key role of Nampt in osteoblastic differentiation from bone marrow stromal cells.

### The Nampt inhibitor FK866 decreased osteoblast formation in C3H10T1/2 cells

**Fig. 2:**
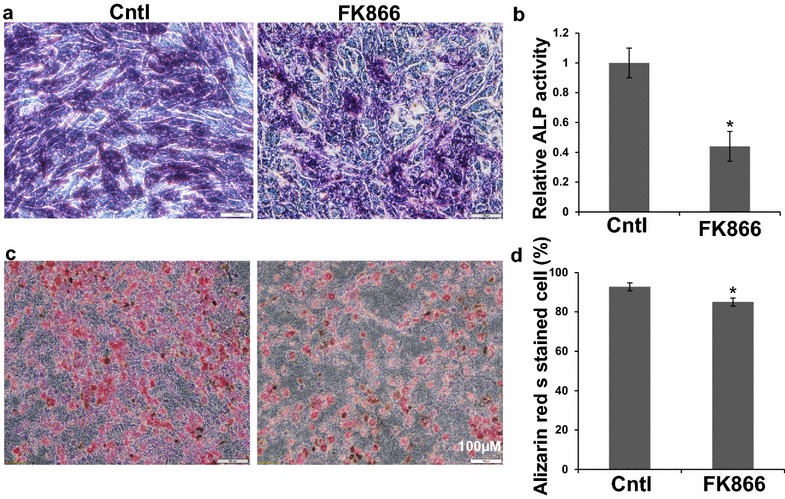
Effects of Nampt enzyme activity inhibition by FK866 on osteoblast differentiation of CH310T1/2 cells. Cells were cultured in 6-well plate in RPMI for 2 days and then were differentiated in OBM with or without FK866 (1 nM) for 14 days. **a** Representative images of ALP stained differentiated osteoblast as described in “Methods”. **b** ALP activity analyzed by PNPP quantification as described in “Methods”. **c** Representative images of the differentiated osteoblast stained by Alizarin Red S as described in “Methods”. **d** Alizarin Red S stained differentiated osteoblast were counted using the Image J in three different view fields and the percentage positive cells over total cells were calculated. n = 3, *p < 0.05 vs FK866 untreated controls

To further strengthen our findings that Nampt could promote osteogenesis in mice (Fig. 1), we used the potent and specific Nampt inhibitor, FK866, to test the effects of Nampt enzymatic activity on osteogenic differentiation in C3H/10T1/2 cells. The C3H10T1/2 cell line was derived from mouse embryonic tissue and can differentiate into osteoblasts under proper stimulation [19], representing a suitable model to study the lineage fate determination of multipotent stem cells. We found that at the non-toxic concentration of 1 nM, FK866 significantly decreased ALP staining (Fig. 2a). ALP activity also decreased significantly: ALP activity in cells differentiated in the presence of 1 nM FK866 was detected as low as 44% of the control cells differentiated without FK866. Consequently, the mineral nodule formation, as demonstrated by Alizarin Red staining, was reduced with the treatment of 1 nM FK866 (Fig. 2c, d). After C3H/10T1/2 cells were cultured in osteogenic media (OBM) for 14 days, the alizarin red staining positive cells were decreased to 73% upon 1 nM FK866 treatment, compared to 91% positive cells in OBM without FK866 treatment (Fig. 2d). These data corroborated that Nampt played a critical role in the osteogenic differentiation from C3H10T1/2 cells.

### Osteogenic differentiation of MC3T3-E1 cells was inhibited by knock-down of Nampt expression

**Fig. 3:**
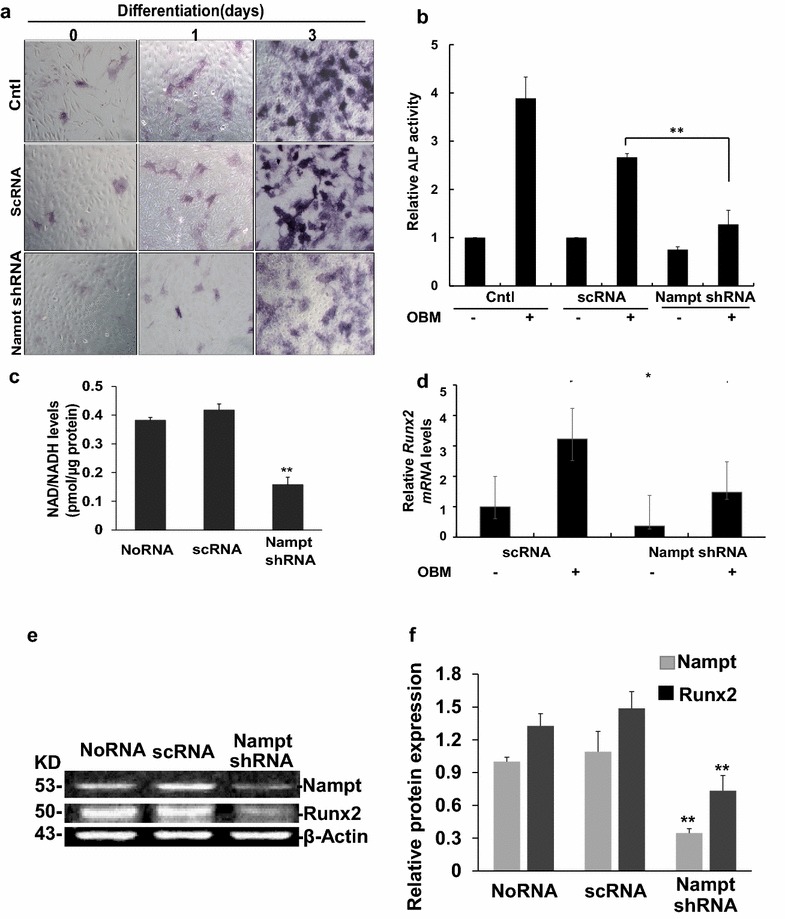
Effects of Nampt-knocking down by shRNA on the differentiation of MC3T3-E1cells. Cells were seeded in 6-well plates and stably transduced with lentivirus with scramble RNA (scRNA) or Nampt shRNA for indicated days. **a** Representative images of MC3T3-E1 cells transduced with no RNA (control), scRNA, and Nampt shRNA. The cells were stained with ALP after 0, 1, and 3 days of differentiation. **b** Activity of ALP was performed after 4 days of differentiation using PNPP as a substrate. **c** Relative total NAD/NADH levels in differentiated MC3T3 cells transduced with lentivirus with scRNA controls, or Nampt shRNA or undifferentiated cells at 3 days using a commercial NADNADH assay kit. **d** Relative RT-PCR quantification of *Runx2* gene expression in differentiated MC3T3-E1 cells transduced with lentivirus with scRNA controls, or Nampt shRNA or undifferentiated cells at 3 days. Runx2 expression was normalized to undifferentiated scRNA control cells. **e** Representative images of Western blotting showing the expression of Nampt and Runx2 in differentiated (at 3 days) MC3T3-E1 cells transduced with lentivirus with scRNA, or Nampt-shRNA. **f** Densitometry analysis of Nampt and Runx2 expression. *Bar* mean ± SD. **p < 0.01 vs. the scRNA control. All experiments were performed in triplicate

To investigate further the role of Nampt on osteogenic differentiation, we generated Nampt deficient MC3T3-E1 cells by transducing the cells with lentivirus packaged with Nampt shRNA. As indicated in Fig. 3e, Nampt expression at the protein level was successfully diminished as compared with the cells transduced with the lentivirus packaged with non-targeting scrambled RNA (scRNA). After 1 and 3 days of osteogenic differentiation, the activity of the osteoblast marker, alkaline phosphatase (ALP), was lower in Nampt knockdown cells as demonstrated by ALP staining (Fig. 3a). After 1 day of differentiation, there was no obvious difference among control cells, cells transduced with scRNA, and Nampt shRNA transduced cells. But after 3 days of differentiation, although there was no difference between control cells and cells transduced with scRNA, both the number of ALP positive cells and stain density were significantly decreased in cells transduced with Nampt shRNA than cells transduced with scRNA and controls. ALP activity assay data also further supported the ALP staining results. In control cells, 4 days after differentiation, the ALP activity was 3.88-fold of undifferentiated cells and in cells transfected with scrambled shRNA, the ALP activity of differentiated cells was 2.66-fold of undifferentiated cells, while in cells transduced with Nampt shRNA, ALP activity was only 1.27-fold of undifferentiated cells, which was significantly lower than in controls cells and scRNA transduced cells (p < 0.01) (Fig. 3b), suggesting that decreased Nampt expression blocked the osteogenic differentiation in MC3T3-E1 cells.

Nampt deficient cells should have lower NAD/NADH levels compared to wild-type cells. Therefore, we assayed intracellular NAD/NADH levels and detected that NAD/NADH levels were significantly decreased in the differentiated MC3T3-E1 cells transduced with Nampt-shRNA lentivirus (Fig. 3c). After 3 days of differentiation for, the NAD/NADH concentration was 0.38 and 0.42 pmol/µg protein in untransduced cells and in cells transduced with control scrambled RNA, respectively. While in cells transduced with Nampt shRNA, the NAD/NADH concentration decreased to 0.16 pmol/µg protein (p < 0.01). The inhibitory effects on osteogenic differentiation were further confirmed by qPCR analysis, which showed that the expression of the key osteoblast transcription factor *Runx2*, was markedly down-regulated (Fig. 3d). In differentiated MC3T3-E1 cells, the *Runx2* mRNA level was 3.23-fold of undifferentiated cells. But in Nampt-shRNA transduced cells, mRNA levels were only 1.47-fold, which was not statistically significant compared with undifferentiated cells (p > 0.05). Western blot analysis was also performed to check the Runx2 expression in differentiated Nampt-deficient MC3T3-E1 cells. After 3 days of differentiation, Runx2 expression in Nampt-deficient cells decreased to 0.48-fold of the cells transduced with scrambled shRNA controls (p < 0.01), in which Nampt was not knocked down (Fig. 3e, f).

### Effects of Runx2 transcription decreased in Nampt deficient MC3T3-E1 cells

**Fig. 4:**
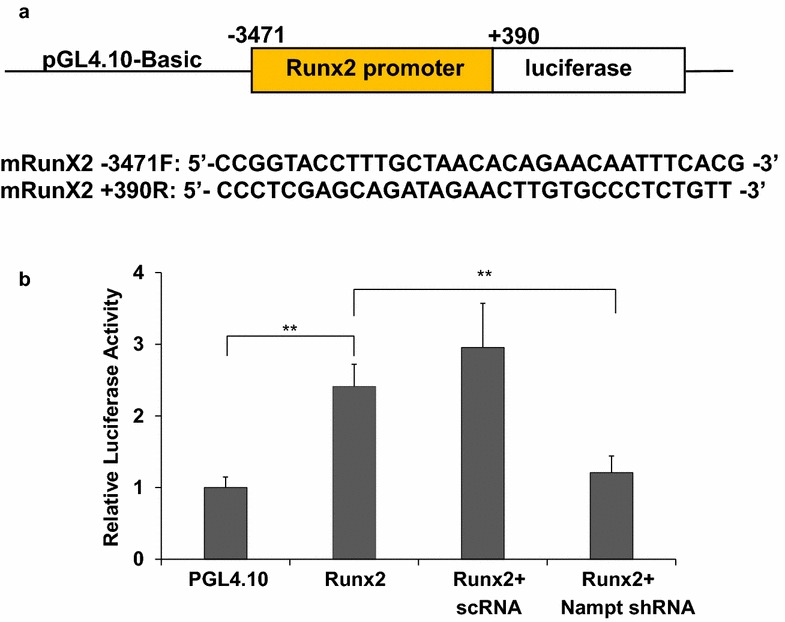
Regulative effects of Nampt on Runx2 transcription in MC3T3-E1 cell differentiation. MC3T3 cells were differentiated for 48 h and then co-transfected with pGL4.10-mRunX2pro firefly luciferase reporter (100 ng), pGL4.75 *Renilla* reporter (4 ng) and either 100 µM of scrambled RNA or Nampt shRNA using Lipofectamine 3000. Transfected MC3T3 cells were incubated for 24 h and luciferase activity was determined using Promega’s Dual-Glo Luciferase Assay Kit. Background corrected firefly luminescence values were normalized with *Renilla* luminescence values. Relative luciferase activities were normalized against differentiated MC3T3 cells transfected with pGL4.10 empty vector. n = 4; *Bars* are mean ± SD. **p < 0.01

To investigate the role of Nampt in *Runx2* transcription, pGL4.10-m*RunX2*pro firefly luciferase reporter plasmid was constructed (Fig. 4a) and luciferase reporter assays were performed. Differentiated MC3T3 cells transfected with 100 ng of m*RunX2* promoter plasmids significantly increased the luciferase activity (Fig. 4b). In m*Runx2* promoter transfected cells, the luciferase activity was 2.41 ± 0.31 fold of untransfected control cells (p < 0.01). While co-transfection with 100 nM Nampt shRNA greatly blocked *Runx2*-promoter luciferase activity. In cells co-transfected with Nampt shRNA and pGL4.10-m*RunX2*pro, luciferase activity was 1.21 ± 0.23 fold of the controls, which was not obviously different from the controls (p = 0.18).

### Nampt regulates Runx2 expression through enhancing histone H3-Lys9 acetylation

**Fig. 5:**
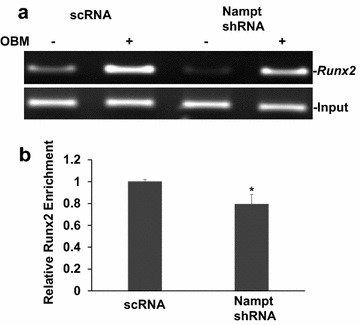
Epigenetic regulation of the Runx2 promoter in Nampt deficient MC3T3-E1 cells. **a** ChIp-PCR of Runx2 promoter following immunoprecipitation with the Acetyl Histone H3 (K9) antibody in scrambled shRNA (scRNA) or Nampt shRNA transduced stable MC3T3-E1 clones with or without differentiation for 72 h. **b** Densitometry analysis of differentiation (OBM)-mediated Runx2 promoter acetylation normalized to input and undifferentiated cells transduced with scrambled shRNA. *Bars* are mean ± SD. Each experiment performed in triplicate. *p < 0.05

The contribution of the H3-Lys 9 acetylation during transcriptional control of *Runx2* was analyzed by knockdown of Nampt during osteoblast differentiation (Fig. 5). ChIP-PCR revealed increased acetylation of the *Runx2* promoter compared with undifferentiated control samples, reaching a 2.77-fold up-regulation (Fig. 5b). As expected, shRNA-mediated knockdown of Nampt attenuated the increase of *Runx2* promoter acetylation associated with osteoblast differentiation. In Nampt shRNA transduced cells, differentiation only increased 2.1-fold of *Runx2*, which is significantly lower than that in scrambled shRNA control cells (p = 0.01). From our data, in Nampt shRNA transduced cells, differentiation increased *Runx2* levels, but significantly lower than control cells or in scrambled shRNA transduced cells. These results suggest that Nampt regulates transcription of *Runx2* in part through regulating H3-Lys9 acetylation.

## Discussion

Human aging is associated with a gradual decline in bone mass and the onset of osteoporosis. Accumulating evidence show that the progressive transition of pluripotent stem cells to the lineage-specific differentiated stages involves dynamic changes in energy demand and in the relative contributions of oxidative and glycolytic metabolic pathways [20–22]. However, a molecular link between energy metabolism and cell differentiation has not been fully elucidated. In the present study, we investigated the roles of Nampt, which is the rate-limiting enzyme in the NAD^+^ salvage pathway, in the osteogenic differentiation of bone marrow stromal cells. We found that in both Nampt-deficient mice and Nampt-deficient cells, osteogenic differentiation was decreased. This decrease is associated with a decrease in NAD+ levels. Furthermore our data suggested that the reduction, in part, is due to the epigenetic inhibition of histone H3-Lys9 acetylation and consequently inhibition of the transcription of *Runx2*, a key transcription factor in osteoblast differentiation.

Characterization of the role of Nampt in osteogenesis has recently attracted more and more attention. Xie et al. [23] found that NAMPT exerts an insulin-like activity as a growth factor for osteoblasts. Decreased Nampt was also suggested to be linked with age-related adipogenesis [13]. However, no studies have shown the role of Nampt in osteogenic differentiation and the molecular mechanisms by which Nampt promotes osteogenesis. To address these questions, we examined osteoblast differentiation in Nampt-deficient (*Nampt* ^+/−^) mice and found that osteoblasts differentiation is diminished in cells derived from bone marrow stromal cells compared to those derived from wild-type mice (Fig. 1). The in vivo studies were supported by in vitro studies showing that both the Nampt enzymatic inhibitor FK866 and Nampt shRNA decreased osteogenic differentiation significantly in murine fibroblast CH310T1/2 and preosteoblastic MC3T3-E1 cells (Figs. 2, 3b, c). These cell lines represent suitable models to study the lineage fate determination of multipotent stem cells [24, 25]. Although Nampt could promote osteogenesis, which may be a target for osteoporosis treatment, it is noticeable that Nampt could act as double-edged swords since its expression is upregulated during inflammation, as NAMPT represents a novel clinical biomarker in acute lung injury [26], rheumatoid arthritis [27], Crohn’s disease [28], and inhibition of Nampt activity attenuates the CLP-induced sepsis in mice [29]. Like some other genes, Nampt’s function is likely dependent on cellular and genetic context.

The osteoblast master regulator Runx2 is predominant-ly a fetal factor and its key role in osteoblast differentiation was convincingly demonstrated in 1997 by Komori et al. [30]. The role of Runx2 is important at two time points: during the exit of pre-osteoblasts from the cell cycle and during the late maturation stages of osteo-blasts [31]. Therefore, the factors that regulate Runx2 will also regulate osteoblast differentiation. We then tested a hypothesis that Nampt may promote osteogenesis through upregulating Runx2 transcription. As expected, our data strongly support our hypothesis (Fig. 3d, e). qPCR data showed that differentiation-induced *Runx2* expression was greatly blocked in Nampt-shRNA stably transduced MC3T3-E1 cells, in which Nampt was significantly knocked down as detected by Western blotting (Fig. 3e). A lower intracellular NAD/NADH levels in differentiated MC3T3-E1 cells was observed (Fig. 3c), suggesting that lower Nampt levels affect the NAD+ synthesis from salvage pathway. Further studies need to address whether the regulation of osteogenesis by Nampt is dependent or independent of its enzyme activity in salvage pathway. Furthermore, the role of Nampt in increasing *Runx2* transcription was also demonstrated by luciferase reporter assays (Fig. 4). In MC3T3-E1 cells, the differentiation-mediated *Runx2* function was nearly totally blocked by knock down of Nampt.

It has been known that promoters of transcribed genes are enriched with hyperacetylation on the N-terminal tail of histone H3 [32]. Acetylation of K9 and K14 in histone H3 is required for the recruitment of transcription factor lid [33], which binds to the promoter causes DNA bending and downstream translocation of the SWItch/sucrose non-fermentable-modified nucleosome, thus allowing the initiation of transcription [34]. Epigenetic mechanisms could be critical for determining the fate of stem cells. In Nampt-deficient MC3T3-E1 cells, differentiation induced *Runx2* levels were significantly lower than in control cells (Fig. 5), together with the results from luciferase assay data, which showed that *Runx2* promoter activity was blocked in Nampt-deficient MC3T3-E1 cells (Fig. 4), suggesting that Nampt promotes osteogenesis partially through inhibiting H3-lys9 acylation (Fig. 5). However, it is well known that Histone H3 has different modifications including acetylation and methylation on different amino acid sites, with each modification conferring distinct effects on gene transcription. Thus, we will investigate this point as well as the more detailed signal transduction networks in the regulation of osteogenic differentiation by Nampt both in vivo and in vitro in future studies.

## Conclusions

In conclusion, our study showed that NAMPT plays a critical role in osteoblastic differentiation. Further, our data indicate that Nampt promotes osteogenesis through the epigenetic regulation of *Runx2* expression and thereby the upregulation of Runx2, a master regulator of osteoblast differentiation. Although more in-depth mechanistic studies are warranted, our findings in this study suggest that Nampt may be a potential therapeutic target of aging-related osteoporosis.

## Abbreviations

NAMPT: nicotinamide phosphoribosyltransferase
PBEF: pre-B-colony-enhancing factor
Q-PCR: quantitative polymerase chain reaction
NAD: nicotinamide adenine dinucleotide
TERT: telomerase reverse transcriptase
Runx2: runt-related transcription factor 2
ALP: alkaline phosphatase
PNPP: P-nitrophenyl phosphate
ChIP: chromatin immunoprecipitation
TRAP: tartrate-resistant acid phosphatase
OBM: osteoblast medium
MSCs: mesenchymal stem cells

## References

[1] Li X, Cao X (2006). BMP signaling and skeletogenesis. Ann NY Acad Sci.

[2] Kong YY, Yoshida H, Sarosi I, Tan HL, Timms E, Capparelli C, Morony S, Oliveira-dos-Santos AJ, Van G, Itie A (1999). OPGL is a key regulator of osteoclastogenesis, lymphocyte development and lymph-node organogenesis. Nature.

[3] Papachroni KK, Karatzas DN, Papavassiliou KA, Basdra EK, Papavassiliou AG (2009). Mechanotransduction in osteoblast regulation and bone disease. Trends Mol Med.

[4] Quarto R, Mastrogiacomo M, Cancedda R, Kutepov SM, Mukhachev V, Lavroukov A, Kon E, Marcacci M (2001). Repair of large bone defects with the use of autologous bone marrow stromal cells. N Engl J Med.

[5] Johnson ML, Rajamannan N (2006). Diseases of Wnt signaling. Rev Endocr Metab Disord.

[6] Keith A (1927). Concerning the origin and nature of osteoblasts. Proc R Soc Med.

[7] Jensen ED, Gopalakrishnan R, Westendorf JJ (2010). Regulation of gene expression in osteoblasts. BioFactors.

[8] Ducy P (2000). Cbfa1: a molecular switch in osteoblast biology. Dev Dyn.

[9] Banerjee C, Javed A, Choi JY, Green J, Rosen V, van Wijnen AJ, Stein JL, Lian JB, Stein GS (2001). Differential regulation of the two principal Runx2/Cbfa1 n-terminal isoforms in response to bone morphogenetic protein-2 during development of the osteoblast phenotype. Endocrinology.

[10] Prince M, Banerjee C, Javed A, Green J, Lian JB, Stein GS, Bodine PV, Komm BS (2001). Expression and regulation of Runx2/Cbfa1 and osteoblast phenotypic markers during the growth and differentiation of human osteoblasts. J Cell Biochem.

[11] Sudhakar S, Li Y, Katz MS, Elango N (2001). Translational regulation is a control point in RUNX2/Cbfa1 gene expression. Biochem Biophys Res Commun.

[12] Revollo JR, Grimm AA, Imai S (2004). The NAD biosynthesis pathway mediated by nicotinamide phosphoribosyltransferase regulates Sir2 activity in mammalian cells. J Biol Chem.

[13] Li Y, He X, Li Y, He J, Anderstam B, Andersson G, Lindgren U (2011). Nicotinamide phosphoribosyltransferase (Nampt) affects the lineage fate determination of mesenchymal stem cells: a possible cause for reduced osteogenesis and increased adipogenesis in older individuals. J Bone Miner Res.

[14] Li Y, He J, He X, Li Y, Lindgren U (2013). Nampt expression increases during osteogenic differentiation of multi- and omnipotent progenitors. Biochem Biophys Res Commun.

[15] Huang P, Riordan SM, Heruth DP, Grigoryev DN, Zhang LQ, Ye SQ (2015). A critical role of nicotinamide phosphoribosyltransferase in human telomerase reverse transcriptase induction by resveratrol in aortic smooth muscle cells. Oncotarget.

[16] Zhang LQ, Van Haandel L, Xiong M, Huang P, Heruth DP, Bi C, Gaedigk R, Jiang X, Li DY, Wyckoff G (2017). Metabolic and molecular insights into an essential role of nicotinamide phosphoribosyltransferase. Cell Death Dis.

[17] Ye SQ, Simon BA, Maloney JP, Zambelli-Weiner A, Gao L, Grant A, Easley RB, McVerry BJ, Tuder RM, Standiford T (2005). Pre-B-cell colony-enhancing factor as a potential novel biomarker in acute lung injury. Am J Respir Crit Care Med.

[18] Tamiya H, Ikeda T, Jeong JH, Saito T, Yano F, Jung YK, Ohba S, Kawaguchi H, Chung UI, Choi JY (2008). Analysis of the Runx2 promoter in osseous and non-osseous cells and identification of HIF2A as a potent transcription activator. Gene.

[19] Katagiri T, Yamaguchi A, Ikeda T, Yoshiki S, Wozney JM, Rosen V, Wang EA, Tanaka H, Omura S, Suda T (1990). The non-osteogenic mouse pluripotent cell line, C3H10T1/2, is induced to differentiate into osteoblastic cells by recombinant human bone morphogenetic protein-2. Biochem Biophys Res Commun.

[20] Cho YM, Kwon S, Pak YK, Seol HW, Choi YM, Park DJ, Park KS, Lee HK (2006). Dynamic changes in mitochondrial biogenesis and antioxidant enzymes during the spontaneous differentiation of human embryonic stem cells. Biochem Biophys Res Commun.

[21] Yanes O, Clark J, Wong DM, Patti GJ, Sanchez-Ruiz A, Benton HP, Trauger SA, Desponts C, Ding S, Siuzdak G (2010). Metabolic oxidation regulates embryonic stem cell differentiation. Nat Chem Biol.

[22] Chen CT, Shih YR, Kuo TK, Lee OK, Wei YH (2008). Coordinated changes of mitochondrial biogenesis and antioxidant enzymes during osteogenic differentiation of human mesenchymal stem cells. Stem Cells.

[23] Xie H, Tang SY, Luo XH, Huang J, Cui RR, Yuan LQ, Zhou HD, Wu XP, Liao EY (2007). Insulin-like effects of visfatin on human osteoblasts. Calcif Tissue Int.

[24] Tanaka SM, Li J, Duncan RL, Yokota H, Burr DB, Turner CH (2003). Effects of broad frequency vibration on cultured osteoblasts. J Biomech.

[25] Lin GL, Hankenson KD (2011). Integration of BMP, Wnt, and notch signaling pathways in osteoblast differentiation. J Cell Biochem.

[26] Liu P, Li H, Cepeda J, Xia Y, Kempf JA, Ye H, Zhang LQ, Ye SQ (2009). Regulation of inflammatory cytokine expression in pulmonary epithelial cells by pre-B-cell colony-enhancing factor via a nonenzymatic and AP-1-dependent mechanism. J Biol Chem.

[27] Presumey J, Courties G, Louis-Plence P, Escriou V, Scherman D, Pers YM, Yssel H, Pene J, Kyburz D, Gay S (2013). Nicotinamide phosphoribosyltransferase/visfatin expression by inflammatory monocytes mediates arthritis pathogenesis. Ann Rheum Dis.

[28] Mesko B, Poliska S, Szegedi A, Szekanecz Z, Palatka K, Papp M, Nagy L (2010). Peripheral blood gene expression patterns discriminate among chronic inflammatory diseases and healthy controls and identify novel targets. BMC Med Genom.

[29] Huang P, Lee MW, Sadrerafi K, Heruth DP, Zhang LQ, Maulik D, Ye SQ (2017). MC-PPEA as a new and more potent inhibitor of CLP-induced sepsis and pulmonary inflammation than FK866. Drug Des Dev Ther.

[30] Komori T, Yagi H, Nomura S, Yamaguchi A, Sasaki K, Deguchi K, Shimizu Y, Bronson RT, Gao YH, Inada M (1997). Targeted disruption of Cbfa1 results in a complete lack of bone formation owing to maturational arrest of osteoblasts. Cell.

[31] Stein GS, Lian JB, van Wijnen AJ, Stein JL, Montecino M, Javed A, Zaidi SK, Young DW, Choi JY, Pockwinse SM (2004). Runx2 control of organization, assembly and activity of the regulatory machinery for skeletal gene expression. Oncogene.

[32] MacDonald VE, Howe LJ (2009). Histone acetylation: where to go and how to get there. Epigenetics.

[33] Agalioti T, Chen G, Thanos D (2002). Deciphering the transcriptional histone acetylation code for a human gene. Cell.

[34] Lomvardas S, Thanos D (2001). Nucleosome sliding via TBP DNA binding in vivo. Cell.

